# Supplementary material for: Tuning of controller parameters using Pythagorean fuzzy similarity measure for stable and time delayed unstable plants
Source: PeerJ Comput Sci. 2023 Aug 10;9:e1504. doi: 10.7717/peerj-cs.1504 (PMC10495962; doi:10.7717/peerj-cs.1504)
Supplement: Supplemental Information 2 [file peerj-cs-09-1504-s002.docx]

|  | **1st Degree** | **2nd Degree** |
| --- | --- | --- |
| $s^{0.1}$ | $\frac{1.6 s + 1}{s + 1.6}$ | $\frac{1.677 s^{2}+ 15.72 s + 1}{s^{2}+ 15.72 s + 1.677}$ |
| $s^{0.2}$ | $\frac{2.566 s + 1}{s + 2.566}$ | $\frac{2.824 s^{2}+ 20.59 s + 1}{s^{2}+ 20.59 s + 2.824}$ |
| $s^{0.3}$ | $\frac{4.136 s + 1}{s + 4.136}$ | $\frac{4.796 s^{2}+ 27.28 s + 1}{s^{2}+ 27.28 s + 4.796}$ |
| $s^{0.4}$ | $\frac{6.724 s + 1}{s + 6.724}$ | $\frac{8.266 s^{2}+ 36.75 s + 1}{s^{2}+ 36.75 s + 8.266}$ |
| $s^{0.5}$ | $\frac{11.1 s + 1}{s + 11.1}$ | $\frac{14.58 s^{2}+ 50.71 s + 1}{s^{2}+ 50.71 s + 14.58}$ |
| $s^{0.6}$ | $\frac{18.82 s + 1}{s + 18.82}$ | $\frac{26.67 s^{2}+ 72.57 s + 1}{s^{2}+ 72.57 s + 26.67}$ |
| $s^{0.7}$ | $\frac{33.53 s + 1}{s + 33.53}$ | $\frac{51.85 s^{2}+ 110.3 s + 1}{s^{2}+ 110.3 s + 51.85}$ |
| $s^{0.8}$ | $\frac{66.13 s + 1}{s + 66.13}$ | $\frac{113.1 s^{2}+ 187.8 s + 1}{s^{2}+ 187.8 s + 113.1}$ |
| $s^{0.9}$ | $\frac{170.9 s + 1}{s + 170.9}$ | $\frac{328.6 s^{2}+ 424.3 s + 1}{s^{2}+ 424.3 s + 328.6}$ |
|  | **3rd Degree** | **4th Degree** |
| $s^{0.1}$ | $\frac{1.757 s^{3}+ 49.67 s^{2}+ 41.97 s + 1}{s^{3}+ 41.97 s^{2}+ 49.67 s + 1.757}$ | $\frac{1.828 s^{4}+ 102.7 s^{3}+ 329.8 s^{2}+ 78.91 s + 1}{s^{4}+ 78.91 s^{3}+ 329.8 s^{2}+ 102.7 s + 1.828}$ |
| $s^{0.2}$ | $\frac{3.101 s^{3}+ 72.7 s^{2}+ 51.88 s + 1}{s^{3}+ 51.88 s^{2}+ 72.7 s + 3.101}$ | $\frac{3.357 s^{4}+ 161 s^{3}+ 453.9 s^{2}+ 95 s + 1}{s^{4}+ 95 s^{3}+ 453.9 s^{2}+ 161 s + 3.357}$ |
| $s^{0.3}$ | $\frac{5.526 s^{3}+ 108 s^{2}+ 65.01 s + 1}{s^{3}+ 65.01 s^{2}+ 108 s + 5.526}$ | $\frac{6.227 s^{4}+ 256.4 s^{3}+ 635 s^{2}+ 116.1 s + 1}{s^{4}+ 116.1 s^{3}+ 635 s^{2}+ 256.4 s + 6.227}$ |
| $s^{0.4}$ | $\frac{10.01 s^{3}+ 163.6 s^{2}+ 83.01 s + 1}{s^{3}+ 83.01 s^{2}+ 163.6 s + 10.01}$ | $\frac{11.74 s^{4}+ 417.1 s^{3}+ 907.9 s^{2}+ 144.6 s + 1}{s^{4}+ 144.6 s^{3}+ 907.9 s^{2}+ 417.1 s + 11.74}$ |
| $s^{0.5}$ | $\frac{18.58 s^{3}+ 254.8 s^{2}+ 108.8 s + 1}{s^{3}+ 108.8 s^{2}+ 254.8 s + 18.58}$ | $\frac{22.72 s^{4}+ 698.8 s^{3}+ 1337 s^{2}+ 185 s + 1}{s^{4}+ 185 s^{3}+ 1337 s^{2}+ 698.8 s + 22.72}$ |
| $s^{0.6}$ | $\frac{35.85 s^{3}+ 413.7 s^{2}+ 148.2 s + 1}{s^{3}+ 148.2 s^{2}+ 413.7 s + 35.85}$ | $\frac{45.73 s^{4}+ 1222 s^{3}+ 2056 s^{2}+ 246.3 s + 1}{s^{4}+ 246.3 s^{3}+ 2056 s^{2}+ 1222 s + 45.73}$ |
| $s^{0.7}$ | $\frac{73.74 s^{3}+ 717.2 s^{2}+ 215 s + 1}{s^{3}+ 215 s^{2}+ 717.2 s + 73.74}$ | $\frac{98.22 s^{4}+ 2287 s^{3}+ 3381 s^{2}+ 349.4 s + 1}{s^{4}+ 349.4 s^{3}+ 3381 s^{2}+ 2287 s + 98.22}$ |
| $s^{0.8}$ | $\frac{170.7 s^{3}+ 1401 s^{2}+ 350.1 s + 1}{s^{3}+ 350.1 s^{2}+ 1401 s + 170.7}$ | $\frac{237.8 s^{4}+ 4833 s^{3}+ 6277 s^{2}+ 557 s + 1}{s^{4}+ 557 s^{3}+ 6277 s^{2}+ 4833 s + 237.8}$ |
| $s^{0.9}$ | $\frac{528.2 s^{3}+ 3662 s^{2}+ 758.6 s + 1}{s^{3}+ 758.6 s^{2}+ 3662 s + 528.2}$ | $\frac{770 s^{4}+ 13690 s^{3}+ 15610 s^{2}+ 1182 s + 1}{s^{4}+ 1182 s^{3}+ 15610 s^{2}+ 13690 s + 770}$ |
